# Drug Responses in Plexiform Neurofibroma Type I (PNF1) Cell Lines Using High-Throughput Data and Combined Effectiveness and Potency

**DOI:** 10.3390/cancers15245811

**Published:** 2023-12-12

**Authors:** Paul O. Zamora, Gabriel Altay, Ulisses Santamaria, Nathan Dwarshuis, Hari Donthi, Chang In Moon, Dana Bakalar, Matthew Zamora

**Affiliations:** 1 MoCo Makers, Gaithersburg, MD 20879, USA; 2 HacDC, Washington, DC 20010, USA; 3 DMV Petri Dish, Rockville, MD 20850, USA; ulisses@dmvpetridish.com; 4 Dan L. Duncan Comprehensive Cancer Center, Department of Molecular and Human Genetics, Baylor College of Medicine, Houston, TX 77030, USA; 5 Lester and Sue Smith Breast Center, Department of Molecular and Human Genetics, Baylor College of Medicine, Houston, TX 77030, USA; 6 National Institute of Neurological Diseases and Stroke, National Institutes of Health, Bethesda, MD 20892, USA

**Keywords:** neurofibromatosis, NF1, high-throughput, plexiform, drug evaluation, in vitro, concentration–response, web tool

## Abstract

**Simple Summary:**

Neurofibromatosis 1 (NF1) is a genetic disorder that predisposes patients to developing nerve sheath tumors that are difficult to treat. There is currently just one drug approved for the treatment of NF1-related inoperable plexiform neurofibromas (for a limited patient population), highlighting the need for further drug discovery in this field. High-throughput screening data are used to guide drug development, but identifying and selecting promising targets can be complex. The aim of our study is to improve the value of high-throughput screening data by combining potency and effectiveness into single-value indices (***S***, ***ΔS***, and ***ΔS mean***), which are used to assess and rank drug sensitivity and drug resistance in cells exposed to potential therapeutic drugs. Our approach with ***S*** indices was applied to cell lines derived from plexiform neurofibromas of patients with *NF1* gene mutations. The use of ***S*** indices provides valuable additional and independent information for discriminating among candidate compounds for follow-up pre-clinical evaluations.

**Abstract:**

*Background*: Neurofibromatosis type 1 (NF1) is a genetic disorder characterized by heterozygous germline *NF1* gene mutations that predispose patients to developing plexiform neurofibromas, which are benign but often disfiguring tumors of the peripheral nerve sheath induced by loss of heterozygosity at the *NF1* locus. These can progress to malignant peripheral nerve sheath tumors (MPNSTs). There are no approved drug treatments for adults with NF1-related inoperable plexiform neurofibromas, and only one drug (selumetinib), which is an FDA-approved targeted therapy for the treatment of symptomatic pediatric plexiform neurofibromas, highlighting the need for additional drug screening and development. In high-throughput screening, the effectiveness of drugs against cell lines is often assessed by measuring in vitro potency (AC50) or the area under the curve (AUC). However, the variability of dose–response curves across drugs and cell lines and the frequency of partial effectiveness suggest that these measures alone fail to provide a full picture of overall efficacy. *Methods*: Using concentration–response data, we combined response effectiveness (EFF) and potency (AC50) into (a) a score characterizing the effect of a compound on a single cell line, ***S*** = log[EFF/AC50], and (b) a relative score, ***ΔS***, characterizing the relative difference between a reference (e.g., non-tumor) and test (tumor) cell line. ***ΔS*** was applied to data from high-throughput screening (HTS) of a drug panel tested on *NF1−/−* tumor cells, using immortalized non-tumor *NF1+/−* cells as a reference. *Results*: We identified drugs with sensitivity, targeting expected pathways, such as MAPK-ERK and PI3K-AKT, as well as serotonin-related targets, among others. The ***ΔS*** technique used here, in tandem with a supplemental ***ΔS*** web tool, simplifies HTS analysis and may provide a springboard for further investigations into drug response in NF1-related cancers. The tool may also prove useful for drug development in a variety of other cancers.

## 1. Introduction

Neurofibromatosis type 1 (NF1) is a rare chronic neurocutaneous disease caused by loss-of-function alterations in the gene *NF1*, encoding the tumor suppressor neurofibromin [1,2]. Via RAS inhibition, *NF1* mutations affect multiple signaling pathways linked to cell survival and proliferation and the development of cancers. NF1 patients often develop neurofibromas, which are benign peripheral nerve sheath tumors. In 8% to 16% of NF1 patients, however, these benign neurofibromas progress to malignant peripheral nerve sheath tumors (MPNSTs) [3,4]. MPNSTs are characterized by the deletion or loss of function of tumor suppressors, including neurofibromin itself, and amplifications or gain of function mutations in several oncogenes, including *MET*, *EGFR*, and other receptor tyrosine kinases. Other deregulated pathways in MPNSTs include the MTOR, HIPPO, WNT, and RAS signaling pathways [5].

Currently, the sole FDA-approved drug treatment for NF1-associated inoperable plexiform neurofibromas is the MEK inhibitor selumetinib [6,7,8], which is indicated for the treatment of pediatric (but not adult) NF1-associated nerve sheath tumors. This deficit highlights the need for different ways to derive promising targets and new drugs.

One drug development technique involves using high-throughput screening (HTS) to assess the effect of an array of drugs on cancer cells and, thereby, identify promising targets for further experimentation. The effective prioritization of drugs depends on the appropriate analysis of the resulting data. Typically, the prioritization of drugs has been quantified through evaluating AC50, or area under the curve (AUC) values. Both techniques have limitations. AC50 is the concentration of a drug that induces half-maximal activity. The relative potency of two compounds can be compared by computing the log of the ratio of two AC50 values (ΔpAC50). For drugs generating complete cytotoxic responses within a given concentration range, AC50 and ΔpAC50 values can effectively be used for comparison. This relationship breaks down, however, when one or both of the drugs being compared are only partially effective, which is a common finding in HTS [9,10,11].

The AUC is the area under the response curve for a cell population over the tested drug concentration range. There are, however, reports of poor concordance of half-maximal inhibitory concentration (IC50) and AUC measures [12] (as well as AC50 and AUC). Guha et al. [13] have also pointed out additional issues involving the use of AUC, which may limit its general utility.

To address these limitations, we developed relative activity scales (*S* indices) for normalizing and measuring drug activity. The S-index combines potency and effectiveness data, facilitating a comparison of the relative activity of compounds across drug classes and cell lines. Normalizing activity by including the maximal response corrects for differences in full or partial drug sensitivity and can be determined without the need to re-fit the underlying concentration–response data to a more complex model [14]. We applied our ***S***-index analysis to a high-throughput screening dataset [15], which screened a panel of 1912 drugs against NF1-patient-derived neurofibroma cell lines and reference peripheral nerve cell lines (see Table 1). The resulting list of drugs to which these cell lines are sensitive or resistant, and the algorithm itself, may prove useful in drug development efforts.

## 2. Materials and Methods

### 2.1. Cell Lines, Data Access, and Extraction 

To assess our analysis algorithm and identify promising targets, we analyzed an HTS dataset produced by Ferrer et al. [15], which reported pharmacologic and genomic profiling of plexiform neurofibroma-derived Schwann cells from NF1 patients and control nerve cells. Proliferation assays were used to screen a panel of 1912 small molecules (the MIPE 4.0 library [17]) against immortalized *NF1*−/−, *NF1*+/−, and *NF1*+/+ cell lines [16], with the fluorescence intensity indicating survival and proliferation after treatment.

Primary files [15] were accessed from Single Agent Screens through Synapse https://doi.org/10.7303/syn4939906 (accessed on 1 May 2023). Access was provided by the Children’s Tumor Foundation through their 2022 Hack4NF project.

The following data were extracted from primary files: cell line name, drug name and target, log AC50 (LAC50, i.e., the log of the active concentration for a half-maximal response in molar concentration), R2 (R^2^, goodness of fit for the 4-parameter logistic regression model) values of dose–response curves, AUC (TAUC, total area under the curve), the asymptote minimum (“INF”, response extrapolated to infinite concentration), and the asymptote maximum (“ZERO”, response extrapolated to zero concentration).

Separately, response data (dat0–dat10) and concentration (C0–C10, in μM) were downloaded and analyzed using a 4-parameter fit in SigmaPlot and with the DREA web tool (see Supplemental Materials). The DREA web tool recreates our final compound rankings using the application’s default filters, based on the data files from Ferrer et al. [15].

### 2.2. Data Analysis

#### 2.2.1. AC50 (Potency) and Relative Potency

The log AC50 values (in M) were converted to AC50 (in μM), which is a commonly used potency measure. The relative potency (∆pAC50) of a compound exposed to two cell lines is defined in Equations (1) and (2). For the data presented in the Results section, the reference (*ref*) cell line was ipnNF95.11C (note that other reference lines can be selected—see our discussion on multiple controls) and the test (*test*) was one of the PNF1 cell lines. Generally, ∆AC50 > 0 indicates that the test cell lines were more sensitive to a compound than the reference cell line.

(1) $$∆AC50=\left[{\log AC50}_{ref}-\log{AC50}_{test}\;\right]=\log\left[\frac{{AC50}_{ref}}{{AC50}_{test}}\right]$$

(2) $$∆pAC50=-\left[{\log AC50}_{ref}-{\log AC50}_{test}\right]=-\log\left[\frac{{AC50}_{ref}}{{AC50}_{test}}\right]$$

In an analogous manner, the AUC, as provided in the original dataset (TAUC. total AUC), was used to determine the relative AUC (∆pAC50) using a method analogous to those described in Equations (1) and (2), except that the appropriate AUC values were used (in place of AC50). As described above, the reference cell line was ipnNF95.11C and the test was one of the PNF1 cell lines.

#### 2.2.2. The ***S*** and ***ΔS*** Indices

We establish an algorithm for creating a single value score ***ΔS***, which compares the relative effect of a given compound between a test cell line and a reference cell line. This is, in turn, generated from ***S***, which incorporates both AC50 (potency) and the effectiveness into a single value for a single compound in a single cell line. Equation (3) defines effectiveness (EFF) from concentration–response curves and is calculated as the response asymptote at the maximum dose concentration (ASM_Max_) minus the response asymptote at the minimum dose concentration (ASM_Min_). These two values (EFF and AC50) were incorporated into a single value ***S***, reflecting both effectiveness and potency (Equation (4)) and, therefore, the overall response for a single compound in a single cell line. The relative value ***ΔS*** assesses change in ***S*** from a reference cell line (***S_ref_****)* to ***S*** from a test cell line (***S_test_***); that is to say (***S_ref_*** − ***S_test_***) (Equation (5)).

(3) $$EFF={ASM}_{Max}-{ASM}_{Min}$$

(4) $$S=\log\left(\frac{EFF}{AC50}\right)$$

(5) $$\Delta S=S_{ref}-S_{test}={\log\left(\frac{EFF}{AC50}\right)}_{ref}-{\log\left(\frac{EFF}{AC50}\right)}_{test}\;\;=\log\left[\left(\frac{EFF_{ref}}{EFF_{test}}\right)\left(\frac{AC50_{test}}{AC50_{ref}}\right)\right]$$

The ***ΔS*** value can be envisioned as a theoretical log molar ratio where both the reference and test are compared in a way that both reports complete responses and correct for partial responses based on the input data. The test response is normalized, relative to the reference. Note that the magnitude of ***S*** depends on the units of response and concentration, but ***ΔS*** is a unitless quantity. This approach is analogous to that described by others and used in receptor pharmacology [10,14,18]. 

The ***ΔS*** score was used to monitor the relative effects of compounds across cell lines and monitor the effects of several compounds in a single cell line. When the compound resulted in a higher ***ΔS*** in the reference cell line than in the test cell line, the test cell line was considered resistant to the compound.

(6) $$Drug\;resistant,\;\Delta S=\left(S_{ref}-S_{test}\;\right)>0$$

When the compound gave a lower ***ΔS*** in the reference cell line than in the test cell line, the test cell line was considered sensitive to the compound.

(7) $$Drug\;sensitive,\;\Delta S=\left(S_{ref}-S_{test}\right)<0$$

Since the tumor cell lines could have different drug sensitivities and harbor mutations that impact different intracellular signaling pathways and processes, we ranked compounds with ***ΔS*** for individual cell lines. Then a consolidated evaluation of cell lines was established as the ***ΔS mean***, which is a simple mean of the ***ΔS*** values for a compound across the PNF1 cell lines.

#### 2.2.3. Prioritization

**Figure 1:**
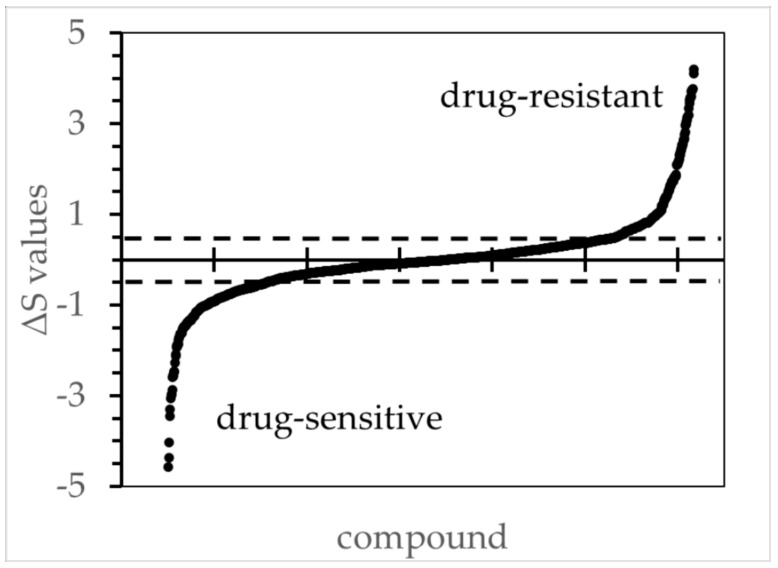
The ***ΔS*** values for all tested compounds comparing ipNF95.6 against the reference cell line ipnNF95.11C. The figure illustrates the order of ***ΔS*** values from low to high based on concentration/response curves, wherein the R^2^ values were ≥ 0.8. The ***ΔS*** thresholds 0.5 and −0.5 are marked with a dotted line, and values beyond those were deemed to be biologically significant during the subsequent candidate selection. Other PNF1 cell lines had similar distributions (not shown).

To further prioritize compounds for a more in-depth follow-up, we selected compounds that generated concentration–response curves with an average R^2^ of at least 0.8 in all four test cell lines. R^2^ values from the reference cell line were similarly set to at least 0.8, and compounds that did not meet this threshold were excluded from the prioritized list, except for cases where there was no concentration–response. For the compounds meeting prioritization criteria, the ***ΔS mean*** and ***ΔS*** variance of the mean were calculated. Compounds displaying drug resistance (***ΔS mean*** > 0.5) or drug sensitivity (***ΔS mean*** < −0.5) were considered potentially biologically meaningful, as they would represent approximately a 3-fold change on an arithmetic scale (Figure 1). An additional threshold of 0.3 was set for the lower boundary of variance of the mean to ensure that the mean values were at least non-inferior to a null response. Non-inferiority can be shown if the difference between the two treatments does not cross a predefined inferiority margin. A threshold of 0.3 would represent approximately a 2-fold difference in an arithmetic scale.

#### 2.2.4. Reference Cell Line and Use of Alternate and Multiple Controls

For our analysis, we primarily used the *NF1*+/− cell line ipnNF95.11C as our reference cell line and the *NF1*−/− plexiform neurofibroma cell lines (PNF1 cells) ipNF05.5-MX, ipNF06.2A, ipNF95.11b C/T, and ipNF95.6 as test lines. The reference ipnNF95.11C cell line is derived from the non-tumor peripheral nerve of an NF1 patient and was selected because individuals with NF1 syndrome have a constitutional heterozygosity for *NF1.* Neurofibromas often arise from cells with additional somatic mutations of *NF1* [14]. Ideal therapies would, therefore, target *NF1*−/− neurofibroma cells, but not background *NF1+*/− cells to yield maximum anti-tumor specificity.

The dataset from Ferrer et. al. also included additional neurofibromin-competent cell lines, specifically human foreskin fibroblasts [HFF] and the Schwann cell lines ipNF02.3 2λ, ipNF02.8. ***ΔS*** calculations using these other control lines can be investigated via the DREA web app.

One way to adapt ***ΔS*** to the comparison of multiple control or tumor cell lines involves selecting one of the control cell lines as the reference and using that reference to evaluate the other control cell lines versus tumor cell lines. ***ΔS*** could be determined using HFF, for example, as the reference for each control cell line and tumor cell line. The ***ΔS means*** of the reference and tumor cell lines would be determined. Then, one can additionally derive the change in the ***ΔS means*** of the reference lines against the tumor cell lines (***ΔΔS***). This approach can be used for a group of different control cell lines or similarly applied to replicates of a single control cell line and multiple tumor cell lines. See supplement Figure S1 on ***ΔΔS*** for an example.

(8) $$\Delta \Delta S={\Delta S\;mean}_{tumor}-{\Delta S\;mean}_{control}$$

## 3. Results

### 3.1. Drug Resistance and Sensitivity Focusing Primarily on a Single PNF1 Cell Line

**Figure 2:**
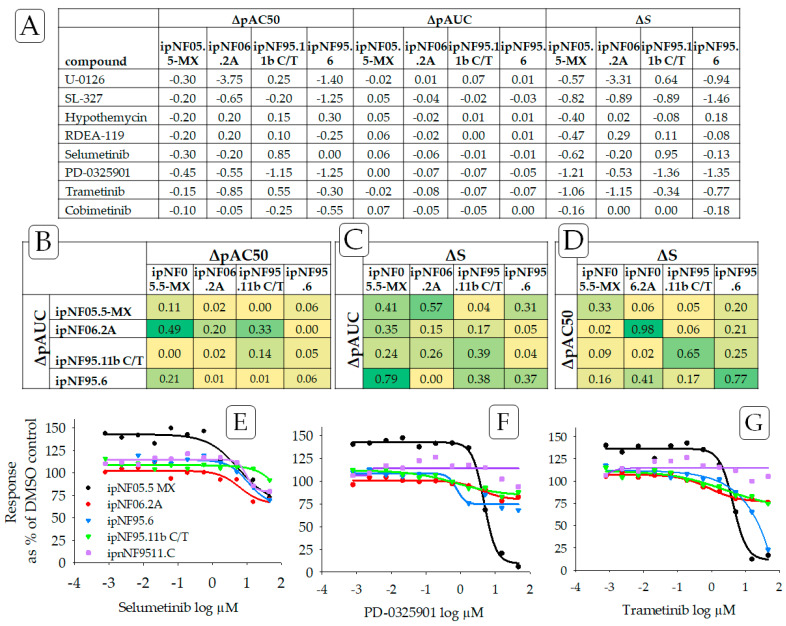
((**A**) **Top panel)** Drug sensitivity of the plexiform neurofibroma cell lines to MEK inhibitors, determined using ΔpAC50, ΔpAUC, and ***ΔS,*** wherein ipnNF95.11C was used as the reference cell line. We compare ***ΔS*** to ΔpAC50 and ΔpAC50, which are frequently used indicators of relative potency. Drug sensitivity responses are negative ***ΔS*** values. ((**B**–**D**) **Middle panel**) Strength of the relationship of ***ΔS*** to ΔpAC50 and ΔpAUC was examined using the square of Pearson’s product-moment correlation coefficient “r” for each pair of cell lines and with color scales applied. The bright green color indicates a high correlation for the relationship, and yellow indicates a low correlation. ((**E**–**G**) **Bottom panel**) Also see graphs that illustrate concentration–response curves for three MEK inhibitors showing drug sensitivity: (**E**) selumetinib, (**F**) PD-0325901 (mirdametinib), and (**G**) trametinib. Purple is used to indicate data from the reference non-tumor cell line, ipnNF9511.C. Note how the concentration–response curves that are shifted below the respective reference lines indicate a relative sensitivity for MEK inhibitors.

To validate the algorithm, we initially evaluated the effects of selumetinib and other MEK inhibitors on one test cell line. We assessed the ***ΔS*** drug sensitivity of ipNF05.5-MX cells (*NF1*−/−) using ipnNF95.11C (*NF1*+/−) as the reference cell line. Using our ***ΔS*** measure, we show that these cells are particularly sensitive to PD-0325901 (mirdametinib) and trametinib, and to a lesser extent, selumetinib (Figure 2). In this case, ***ΔS*** scores appear to have reasonable concordance with the concentration–response curves. These results agree with clinical trial results, showing significant reductions in plexiform tumor volume after treatment with mirdametinib or trametinib [19,20,21,22].

Since ***ΔS*** may be considered an alternative to AC50 and AUC-based methods, the Pearson product-moment correlation was used to compare outcomes from ***ΔS***, ΔpAC50, and ΔpAUC (Figure 2B–D) from three MEK inhibitors (selumetinib, PD-0325901, and trametinib). No strong correlation was found for any pair of endpoints (ΔpAC50 and ΔpAUC, ΔpAC50 and ***ΔS***, ΔpAUC and ***ΔS***) across all plexiform neurofibroma cell lines. A moderate correlation was found, however, when comparing ΔpAC50 and ***ΔS*** for individual cell lines. This outcome could be largely anticipated as those endpoints measure different, albeit interrelated, components of the concentration–response curve (also see Supplemental Table S1). As an extension, no strong correlation was found when monitoring rank outcomes (see Section 3.4—Comparing Ranking Methodologies) from the ***ΔS mean***, ΔpAC50 mean, and ΔpAUC mean, again suggesting that each endpoint is monitoring a different aspect of the concentration–response curves.

We also note that multiple controls can be used in a single analysis by using Equation (8). See Supplemental Figure S1 for an example.

**Figure 3:**
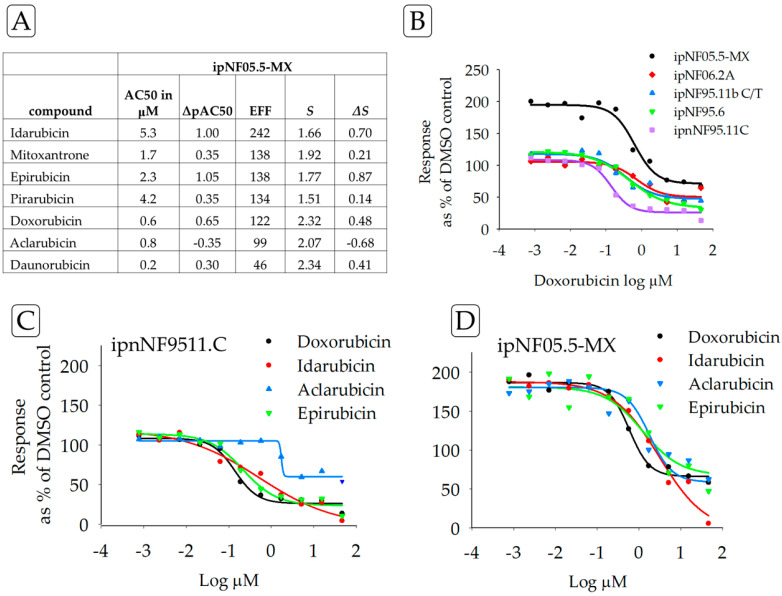
(**A**) Drug resistance of the ipNF05.5-MX cell line by ***ΔS*** for TOP2A inhibitors with ipnNF95.11C as the reference cell line, compared to AC50 and ΔpAC50, which are frequently used as potency indicators. Drug-resistant responses are positive ***ΔS*** values. Using the square of Pearson’s product-moment correlation coefficient “r”, a strong correlation was found between ***ΔS*** and ΔpAC50 (0.9). (**B**) The graph illustrates the concentration–response curves for one of the test compounds, doxorubicin, in all the test cell lines. The purple line is the reference, ipnNF95.11C. (**C**) Concentration–response curves for the reference cell line ipnNF95.11C treated with different TOP2A inhibitors; compared with curves on the right. (**D**) Concentration–response curves for the plexiform neurofibroma cell line ipNF05.5-MX treated with different TOP2A inhibitors; compared with curves on the left. Collectively, the table and graphs permit a comparison of different parameters for one cell line and the effects of multiple drugs.

Individual low-concentration responses higher than 100% of the DMSO control were indicative of drug-stimulated growth, as found for ipNF05.5-MX treated with MEK inhibitors and doxorubicin (Figure 2 and Figure 3). The underlying reason that some drugs display asymptote maximum values above 100% of the DMSO control in Figure 2 and Figure 3 (particularly in ipNF05.5-MX) is not clearly understood. It is possible that these low-concentration responses are due to an unknown experimental variability, or some inherent cell line-to-cell line drug response variability. Supplement Figure S2 compares the histograms of the upper asymptotes from all the plexiform cell lines, including ipNF05.5-MX. All the histograms demonstrated a bell-shaped curve with broadly similar centers. This suggests that it is unlikely that there was a gross plating error or focal cell number error that led to the apparent stimulatory response. It is possible that there is some inherent cell line-to-cell line cause of the low-dose drug response affecting ipNF05.5-MX, which might include hormesis among other explanations.

In contrast to compounds inducing sensitivity, ipNF05.5-MX cells showed resistance to a panel of TOP2A inhibitors, compared to the response of the control ipnNF95.11C cell line (Figure 3). Furthermore, drug resistance was found for 6/7 anthracyclines in ipNF05.5-MX cells. These results were also observed in other PNF1 cell lines when examining the concentration–response curves. Analyses of drugs against a single cell line may be useful when developing precision medicine treatments for tumors that share genetic or proteomic similarities [23].

### 3.2. Drug Assessment by Signaling Pathway

In some cases, researchers may want to test whether a molecular signaling pathway as a whole is a promising target for drug discovery. To evaluate drugs across PNF1 cell lines, we used ***ΔS mean***, a simple mean of the ***ΔS*** values for all four PNF1 cell lines. Table 2 shows the ***ΔS mean*** for compounds known to affect the PI3K/AKT/MTOR signaling pathway. The top hits in this pathway are GNE-490, triciribine phosphate, and WYE-354, which are known inhibitors of PI3K, AKT, and MTOR, respectively.

**Figure 4:**
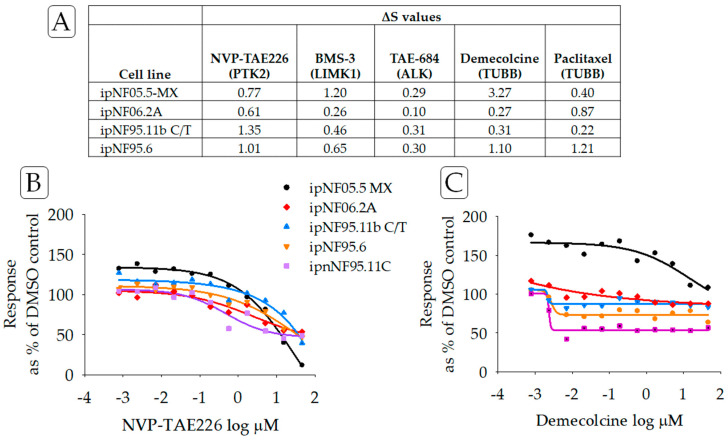
(**A**) ***ΔS*** scores for compounds targeting non-RAS proteins and reportedly binding or associated with neurofibromin. PTK2 (focal adhesion protein, FAK), TUBB (tubulin), LIMK1 (LIM Domain Kinase 1), and ALK (anaplastic lymphoma kinase). (**B**) Concentration–response curves for NVP-TAE226, an inhibitor of FAK, and (**C**) demecolcine, an inhibitor of tubulin. Lines in purple indicated data from the reference cell line ipnNF95.11C.

Additionally, PNF1 tumor cell lines as a group were largely resistant to compounds that target non-RAS-binding partners of neurofibromin [24,25]. Concentration–response curves for two compounds that impact such non-RAS binding partners are found in Figure 4. These binding partners include tubulin [25], HTR6, the focal adhesion protein (FAK) [24,26], and LIMK2 [27]. Here, NVP-TAE226, a FAK inhibitor, and BMS-3, a LIMK1/2 inhibitor, each displayed drug-resistance in PNF1 cell lines.

Arranging the results by the ***ΔS mean*** score and signaling pathway target may be useful in identifying promising drugs for follow-up (see Table 2). In this regard, AKT inhibitors triciribine phosphate and A-443654 may be of downstream interest.

### 3.3. Discussion of Prioritized Compounds

**Figure 5:**
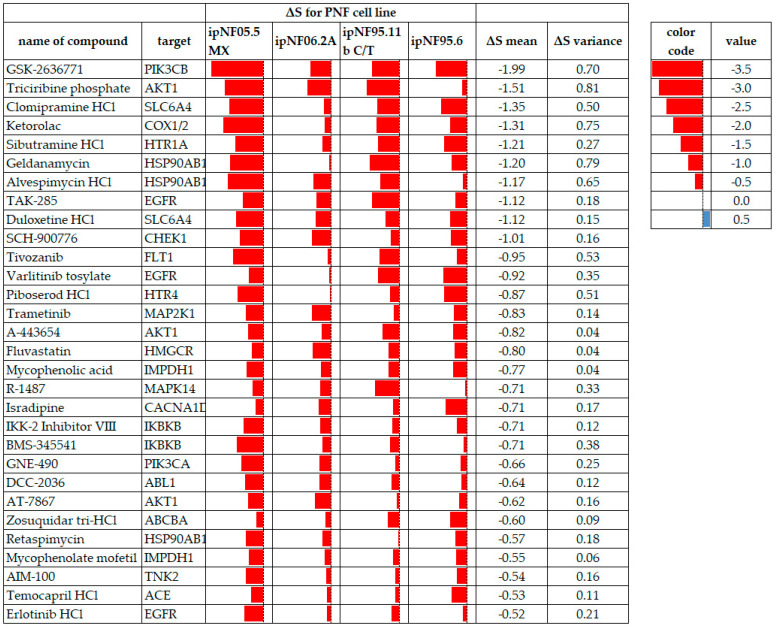
PNF tumor cell lines displaying drug sensitivity to test compounds meeting the ***ΔS mean*** threshold and variance criteria, where all cell lines had data. Red data bars indicate the ***ΔS*** for each cell line with lower values (more sensitivity) to the left in each cell. ***ΔS mean*** values are arranged from the most sensitive to the least sensitive. A red color indicates compound sensitivity, and blue indicates compound resistance.

**Figure 6:**
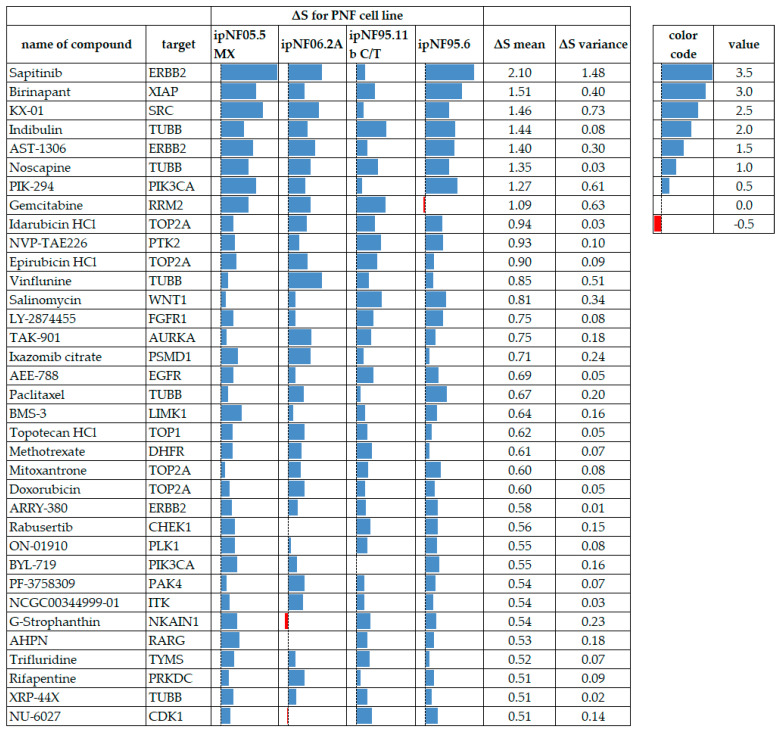
PNF1 tumor cell lines displaying drug-resistance to test compounds meeting the ***ΔS mean*** threshold and variance criteria, where all cell lines had data. Blue data bars indicate the ***ΔS*** for each cell line with higher values (more resistance) to the right in each cell. Red bars indicate drug sensitivity. ***ΔS mean*** values are arranged from the most resistant at the top to the least resistant at the bottom. A red color indicates compound sensitivity, and blue indicates compound resistance.

In this section, we will discuss a subset of the drugs identified as ‘high-sensitivity’ or ‘resistance’. Analyses of other compounds are detailed in Figure 5 and Figure 6 and can be explored via the DREA web tool (see Supplemental Materials). Prioritized compounds have an average R^2^ of at least 0.8 in all four test cell lines and display drug resistance (***ΔS mean*** > 0.5) or drug sensitivity (***ΔS mean*** < −0.5), as described in the methods section. For the current analysis, we compared the response of each cell line (*NF1*−/−) to that of the ipnNF95.11C (*NF1*+/−) cell line, but responses can also be calculated using several other control lines in DREA.

#### 3.3.1. MEK Inhibitors

Several MEK inhibitors have been used in clinical trials for NF1-related tumors, including the FDA-approved drug selumetinib [7,8]. Our analysis identified trametinib as a prioritized compound, to which PNF1 cell lines as a group were sensitive. Trametinib shows promising results in clinical trials, significantly reducing the neurofibroma tumor size [22,28]. While selumetinib was not prioritized by ***ΔS mean***, some cell lines showed sensitivity to it, suggesting it may be effective in specific tumors or cells therein. In vivo, different neurofibromas, even in the same individual, can carry different somatic *NF1* mutations [29]. This can affect both RAS and non-RAS cell responses. Alternatively, the differences could reflect a more robust compensatory increase in pMEK levels, indicating the occurrence of reactivation of the MAPK pathway [25].

#### 3.3.2. PI3K and AKT1 Inhibitors

The most pronounced drug sensitivity across PNF1 cell lines was to the class I PI3KCB inhibitor GSK-2636771. GSK-2636771 is effective in PTEN-deficient tumors [30,31], presumably compensating for the upregulation of the PI3K/AKT pathway engendered by the loss of PTEN inhibition. PTEN pathway alterations have been implicated in early NF1-associated tumorigenesis [32], with marked PTEN reductions found in MPNSTs compared to both neurofibromas and normal nerve tissue [33,34]. Another PI3K-targeted compound identified in our sensitivity analysis was GNE-490, an experimental, pan-PI3K inhibitor that would be expected to inhibit PI3KCB and PI3KCA [35]. GNE-490 is effective against mouse xenograft models of breast and prostate cancers [35].

In contrast, drug resistance was found for two PI3K inhibitors (see Figure 6): PIK-294, an inhibitor that is somewhat selective for the PI3K catalytic subunit p110δ [36], and to a lesser extent, BYL-719, which is selective for the p110α subunit [37]. The result for BYL-719 is unexpected, given the sensitivity of the cells to GNE-490, another p110α inhibitor. This may suggest differing structure–activity relationships [38] between these compounds and GNE-490 [35].

Acting downstream of PI3K, cell lines were sensitive to three AKT1 inhibitors (triciribine phosphate, A-443654, and AT-7867). Triciribine phosphate is the only one of these compounds that has reached the investigational stage of drug development [39]. The identification of AKT-targeting compounds is particularly interesting, given that in MPNSTs neither AKT nor mTORC2 are required for tumor remission [40]. This may reflect important differences in pathway utilization between MPNSTs and PNF1. No AKT-targeting drugs were resistant in our analysis.

#### 3.3.3. *TOP2A* and *CHEK1* Gene Product Inhibitors

*TOP2A*, one of the top 20 genes upregulated in MPNST [41], encodes the enzyme DNA topoisomerase II alpha, which controls and alters the topologic states of DNA during transcription. Matching this observation, PNF1 tumor cell lines displayed drug-resistance to anthracycline topoisomerase inhibitors, e.g., idarubicin, epirubicin, mitoxantrone, and doxorubicin. Finding anthracycline resistance in PNF1 cell lines suggests that this resistance develops early in the tumorigenesis cascade and may later contribute to the poor therapeutic response of doxorubicin in unresectable MPNSTs [42].

DNA topoisomerase II alpha activates cell cycle progression from the G2 to the M phase by inhibiting CHK1 phosphorylation [43] (CHK1, checkpoint kinase 1, is the protein product of the *CHEK1* gene), promoting the epithelial-to-mesenchymal transition and cancer cell invasion. CHK1 is required for checkpoint-mediated cell cycle arrest and is upregulated in MPNST, compared to neurofibromas [44]. We found that cells were sensitive to SCH-900776, which is a selective CHK1 inhibitor. However, these cells were resistant to the CHK1 inhibitor rabusertib. This observation also indicates that there may be an important role in the structure–activity relationships of these compounds and their target. In addition to *CHEK1*, neurofibromas frequently harbor alterations in *PRKDC* [45], which may contribute to resistance.

#### 3.3.4. Heat Shock Proteins

We found sensitivity to three drugs targeting HSP90AB1 (geldanamycin, alvespimycin HCl, and retaspimycin). Given that the loss of neurofibromin activates HSF1 to promote carcinogenesis [46], a finding that plexiform neurofibroma cell lines are sensitive to HSP90AB1 inhibitors is not entirely unexpected, and the potential for heat shock protein inhibitors as NF1 treatments has been suggested [47]. Combined treatment with mTOR and HSP90 inhibitors in vitro led to a decrease in LD50 in human and murine MPNST cell lines compared to a human fibroblast cell line (IMR90), and in vivo, to an increase in survival in tumor-bearing Nf1/p53 mutant mice [48]. While geldanamycin is too toxic to be used clinically, geldanamycin analogs and other HSP90 inhibitors may be good targets for the downstream combination drug evaluation.

#### 3.3.5. Serotonin Modulators

A subset of four prioritized drugs act via serotonin signaling. This is notable in that several antidepressants have carcinostatic effects [49]. Two serotonin reuptake inhibitors (the tricyclic antidepressant clomipramine and the serotonin-noradrenaline reuptake inhibitor duloxetine) were prioritized based on ***ΔS mean***. Clomipramine interferes with autophagic flux in HeLa cells and inhibits growth and “stemness” in lung cancer via its metabolite desmethylclomipramine [50]. Duloxetine is a dual serotonin/norepinephrine reuptake inhibitor that enhances tumor necrosis factor-related apoptosis-inducing ligand (TRAIL) apoptosis in tumor cells [51].

Two serotonin receptor antagonists (piboserod and sibutramine) were also prioritized. Piboserod is a selective antagonist of the G-protein-coupled HTR4 serotonin receptor [52]. Sibutramine, in vivo, is a norepinephrine, serotonin, and dopamine reuptake inhibitor; it has an affinity for several monoaminergic receptors, including HTR1. Both HTR1 and HTR4 can, in some tumor types, act through both the MAPK-ERK pathway and the PI3K-AKT-MTOR pathway [53].

#### 3.3.6. Non-Prioritized Compounds of Interest

We prioritized drug responses that had an average R^2^ of at least 0.8 in all four test cell lines and that displayed drug resistance (***ΔS mean*** > 0.5) or drug sensitivity (***ΔS mean*** < −0.5). There were, however, drug responses in the original panel that represented missing values or responses in fewer than four cell lines. Some of the drugs with the highest ***ΔS mean*** drug-sensitive scores were not included in the final lists due to a single cell line with missing or inconsistent data. However, these compounds (secoisolariciresinol and verteporfin, for example) may also be of interest in follow-up studies.

### 3.4. Comparing Ranking Methodologies

**Figure 7:**
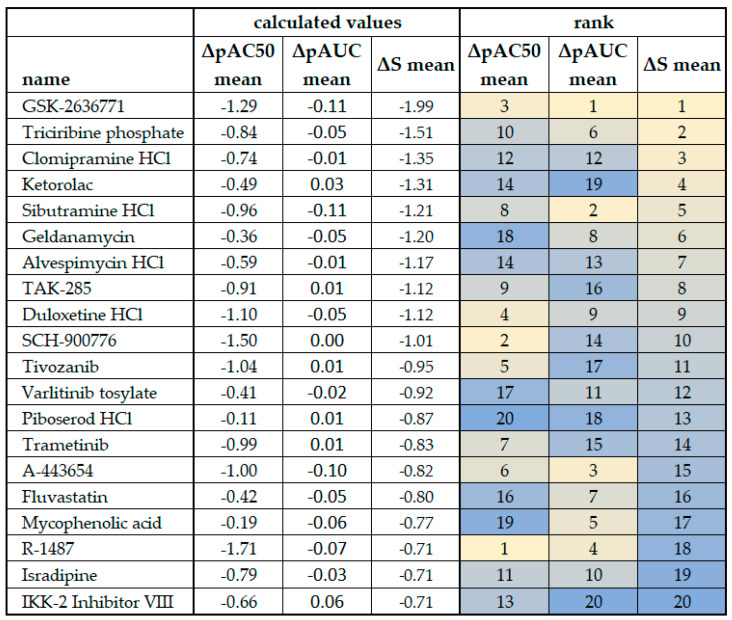
Comparison of ΔpAC50 mean, ΔpAUC mean, and ***ΔS*** mean scores from plexiform neurofibroma cell lines. The cell line ipnNF9511.C derived from the non-tumor nerve was used as the reference in the calculations. Outcomes for ***ΔS mean*** (see Figure 5) are ranked and color-coded to facilitate comparison, and the rank is compared to that derived from the ΔpAC50 mean and ΔpAUC mean. Bright yellow indicates the top ranking for each column/calculation method and dark blue indicates the bottom ranking.

Figure 7 provides a relative ranking of test compounds anchored on ***ΔS means***, with comparisons to ΔpAC50 means and ΔpAUC means. While there were some similarities in rankings, by and large, the rankings across compounds showed little concordance in paired comparisons of ΔpAC50 means, ΔpAUC means, and ***ΔS means***. This is similar to outcomes found in Figure 2 for ΔpAC50, ΔpAUC, and ***ΔS***. Since these endpoints are derived from the same test system and concentration–response curves, the lack of concordance suggests that ΔpAC50, ΔpAUC, and ***ΔS*** measure different aspects of concentration–response curve information.

AC50 and ΔpAC50 are used to monitor changes in drug potency. They do not take into account response effectiveness, which is a second dimension of a concentration–response curve, and can be misleading when comparing drug responses that display full effectiveness to those with partial effectiveness.

The AUC is based on segmenting the entire two-dimensional area underneath the curve and then summing all the sub-values, thereby providing an aggregate measure that considers potency and effectiveness. Differences in the AUC can imply changes in drug sensitivity. Such aggregate values may not always be desirable and may complicate the interpretation of ΔpAUC because, as pointed out by Guha et al. [13], the AUC for a compound exhibiting a shift in potency but no shift in efficacy can be the same as (or similar to) the AUC for a compound exhibiting a shift in efficacy but not a shift in potency.

The ***S*** indices (***S*** and ***ΔS***) incorporate effectiveness and potency and normalize activity through the inclusion of EFF, which is the response effectiveness. This corrects for differences in full or partial drug sensitivity. ***ΔS*** can infer the sensitivity (or resistance) of a given drug in a tumor cell line(s) (a proxy for tumors) relative to a reference cell line (a proxy for normal tissue). Compounds with high sensitivity in plexiform neurofibroma cell lines based on the ***ΔS*** or ***ΔS mean*** would be good candidates for follow-on confirmatory and/or in vivo studies. It is likely preferable to investigate treatments for non-malignant plexiform neurofibromas, which have high tumor sensitivity relative to normal tissues, as a patient may receive protracted drug treatment.

## 4. Conclusions

In this study, AC50 and EFF were combined into a single value (***S***) to furnish system-independent ratios (***ΔS*** and ***ΔS mean***), used to assess the relative drug sensitivity and drug resistance of a panel of compounds. The combination of EFF and AC50 into a single value considers the heterogeneous effects of partial and full drug responses. Using our algorithm, we recapitulate the identification of MEK inhibitors as potential therapeutics for NF1-related tumors and identify other compounds to which PNF1 cell lines are particularly sensitive or resistant. We suggest that the algorithms behind the Drug Response Evaluation and Assessment (DREA) web tool (available at: https://nf.mocomakers.com) can also be used in both primary cell cultures and other cell models, including induced pluripotent stem cells (iPSCs) [54]. Given high-throughput data, the algorithm can be applied to any cancer model as needed. For example, the used dataset [15] had other cell lines, such as human foreskin fibroblasts (HFFs) that may be useful in other styles of analysis.

Compounds with partial or low efficacy can represent important modulators of biological activity and may be particularly difficult to recognize based on AC50 analysis only. The use of ***ΔS*** may help to identify promising compounds that display partial effectiveness. The use of ***ΔS mean*** outcomes from multiple cell lines, as used here, may help in addressing drugs that generate partial responses in vitro and help address the known heterogeneity found in tumor cells and tumor cell lines in general [55,56,57]. DREA can be used to assess candidate compounds individually by the cell line, as in Figure 5 and Figure 6. This allows an analysis to reflect the molecular heterogeneity of different cell lines. In the development of treatments, wide applicability of the treatment is vital. Therefore, we also assess the ***ΔS mean*** and variance values, reflecting responses of PNF1 cell lines as a group, with the goal of assuring biological relevance and approaching response uniformity across PNF1 cell lines.

The current study focused on the use of single agent screens; however, it is possible that the ***S*** indices could be useful to guide the examination of drug combinations using a modification of the IDACombo method [58] and/or SynergyFinder 3.0 [59]. IDACombo is based on independent drug action (IDA), where the therapeutic benefit is derived from the single most effective drug in a drug combination. SynergyFinder 3.0 incorporates a parametric synergy scoring model and multi-dimensional synergy of combinations (MuSyC), which provides the users with the possibility to distinguish whether the identified synergy is due to enhanced potency and/or efficacy of the single agents.

The use of ***S*** indices, particularly ***ΔS mean*** when data are available for multiple cell lines with a common reference line, can be useful in the high-throughput screening assessment of potentially therapeutical useful compounds for the treatment of NF1-related tumors and other cancers. When prioritizing research for a rare disease such as NF1, a cross-compound analysis such as this may be of utility for focusing on the promising mechanisms of action, as well as investigational therapeutics. Our results provide both an affirmation of already approved or developing therapeutics, but also serve as the basis for further pre-clinical evaluations of high-scoring but sparsely explored compounds.

It is also essential to increase to robustness of our method to incorporate multiple control cell lines within the analysis when possible. In this analysis, we used a limited dataset of plexiform neurofibroma cell lines, precluding the use of multiple controls in the current work. However, in fields like breast cancer, where there are more known control lines, an ***ΔΔS*** analysis with multiple controls could likely be done.

It is envisioned that ***ΔS*** could be used as part of an overall orthogonal approach to drug evaluation and selection. Orthogonal approaches combined with ***ΔS*** could help increase the predictive value of in vitro concentration–response studies and, thereby, reduce the cost and time constraints of follow-on in vivo studies and pre-clinical drug development. With an orthogonal approach, ***ΔS*** may also be able to provide a different perspective, if not clarity, on known issues related to the individual use of AC50, effectiveness, or AUC.

## Figures and Tables

**Table 1:** Description of immortalized human cell lines used in the study.

Human Cell Lines			
Cell Line	Immortalized	Tissue Source	Neurofibromin Status
ipnNF95.11C *	yes	Peripheral nerve (non-tumor)	+/−
ipNF06.2A	yes	Plexiform Neurofibroma (PNF1)	−/−
ipNF95.6 *	yes	Plexiform Neurofibroma (PNF1)	−/−
ipNF95.11b C/T	yes	Plexiform Neurofibroma (PNF1)	−/−
ipNF05.5 Mixed Clone (ipNF05.5-MX)	yes	Plexiform Neurofibroma (PNF1)	−/−

* A recent analysis (M.R. Wallace, personal communication) has suggested that the cell lines ipnNF95.11C and ipNF95.6 may consist of a mixed population of homozygous *NF1*−/− and heterozygous *NF1*+/− cells, although the initial testing [15,16] reported them as in the table above and we analyzed them as such.

**Table 2:** ***ΔS*** drug sensitivity for compounds affecting the PI3K/AKT/mTOR pathway, sorted by the ***ΔS mean*** from all four PNF1 cell lines.

PI3KCA			AKT1			MTOR		
Compound	*ΔS Mean*	*ΔS* Variance	Compound	*ΔS Mean*	*ΔS* Variance	Compound	*ΔS Mean*	*ΔS* Variance
GNE-490	−0.66	0.25	Triciribine-PO4	−1.51	0.81	WYE-354	−0.53	1.15
NIBR-17	−0.48	0.14	A-443654	−0.82	0.04	AZD-2014	−0.34	0.24
PIK-90	−0.45	0.01	AT-7867	−0.62	0.16	GDC-0980	−0.24	0.14
BKM-120	−0.33	0.47	AZD-5363	−0.36	0.10	KU-0063794	−0.23	0.04
PF-04691502	−0.31	0.20	GDC-0068	−0.34	0.12	AZD-8055	−0.22	0.38
GNE-493	−0.28	0.08	H-89	−0.26	0.12	WAY-600	−0.20	0.19
PIK-93	−0.28	0.23	MK-2206	−0.13	0.02	PP-242	−0.20	0.89

## Data Availability

In the provided Supplementary Material. This tool and our algorithm are available on https://github.com/MoCoMakers/nf_streamlit/ (accessed on 8 September 2023). Quantitative High-Throughput Screening data are available for the MIPE4.0 library of small molecules from https://www.synapse.org/#!Synapse:syn5522627 (accessed on 1 May 2023).

## Supplementary Materials

The following supporting information can be downloaded at: https://www.mdpi.com/article/10.3390/cancers15245811/s1, Table S1: Simulated effect of changing EFF and AC50 for a number of model compounds; Figure S1: ***ΔΔS*** outcomes from a series of compounds targeting MEK, and targeting JAK2; Figure S2: Histogram of maximum (upper) asymptotes for all tested compounds by target plexiform neurofibroma cell line. An exploratory Drug Response Evaluation and Assessment (DREA) Web Tool was developed in the Python programming language for our methods. This tool can be accessed at https://nf.mocomakers.com and it includes data visualizations, our derived fields, as well as additional filters and cell lines for expanded investigation potential. For more tips on using this tool, please see: https://github.com/MoCoMakers/nf_streamlit/wiki.
