# Supplementary material for: Drug Responses in Plexiform Neurofibroma Type I (PNF1) Cell Lines Using High-Throughput Data and Combined Effectiveness and Potency
Source: Cancers (Basel). 2023 Dec 12;15(24):5811. doi: 10.3390/cancers15245811 (PMC10742026; doi:10.3390/cancers15245811)
Supplement: Supplementary file 1 [file cancers-15-05811-s001.zip › cancers-2757704-supplementary.pdf]

Supplement Table S1. Simulated effect of changing EFF and AC50 for a number of model compounds. Note that when EFF is 100 (full response) the ΔpAC50 and the ***ΔS*** have identical values. When EFF varies and AC50 is constant ***ΔS*** can discriminate between the compounds whereas ΔpAC50 cannot. When EFF and AC50 both vary, both ΔpAC50 and the ***ΔS*** can discriminate between compounds, however, ***ΔS*** has a larger numerical spread for partial response data. This results from combining EFF and AC50 into a single value, ***S***. Compounds with full responses in their concentration response curves, (when EFF is 100%) produce linear, lateral displacement of their pAC50 values along the concentration axis of their concentration-response curve in keeping with the classical use of AC50. This allows direct comparison of pAC50 values in the form of ΔpAC50. Under those conditions, ***ΔS*** as described in the current study will have values similar to those produced using ΔpAC50. In contrast, compounds generating partial responses in their concentration response curves have both lateral and vertical displacement of their pAC50 values producing distinctly nonlinear values along the concentration axis. This is particularly the case for compounds that have partial responses, depressed maxima and similar or AC50 values. This can make discrimination between compound responses difficult. Since ***ΔS*** is based on combining EFF and AC50 into a single value (**S**), it can more effectively discriminate between the responses of different compounds whether they generate full responses or partial responses.

**EFF constant and AC50 varies**						
**compound**	**EFF***	**AC50**	*S*	**pAC50**	***ΔS***	**ΔpAC50**
A	**100**	40	-0.40	-1.60	**-0.22**	**-0.22**
C	**100**	32	-0.49	-1.51	**-0.12**	**-0.12**
E	**100**	24	-0.62	-1.38	**0.00**	**0.00**
G	**100**	16	-0.80	-1.20	**0.18**	**0.18**
I	**100**	8	-1.10	-0.90	**0.48**	**0.48**
REFERENCE	*100*	*24*	*-0.62*	*-1.38*	---	---
**EFF varies and AC50 constant**						
**compound**	**EFF***	**AC50**	*S*	**pAC50**	***ΔS***	**ΔpAC50**
AA	10	**24**	0.38	-1.38	-0.68	**0.00**
CC	30	**24**	-0.10	-1.38	-0.20	**0.00**
EE	50	**24**	-0.32	-1.38	0.02	**0.00**
GG	70	**24**	-0.46	-1.38	0.16	**0.00**
II	90	**24**	-0.57	-1.38	0.27	**0.00**
REFERENCE	*50*	*24*	*-0.30*	*-1.38*	---	---
**EFF varies and AC50 varies**						
**compound**	**EFF***	**AC50**	*S*	**pAC50**	***ΔS***	**ΔpAC50**
AAA	10	40	0.60	-1.60	**-0.90**	***-0.22***
CCC	30	32	0.03	-1.51	**-0.33**	***-0.12***
EEE	50	24	-0.32	-1.38	**0.02**	***0.00***
GGG	70	16	-0.64	-1.20	**0.34**	***0.18***
III	90	8	-1.05	-0.90	**0.75**	***0.48***
REFERENCE	*50*	*24*	*-0.30*	*-1.38*	---	---

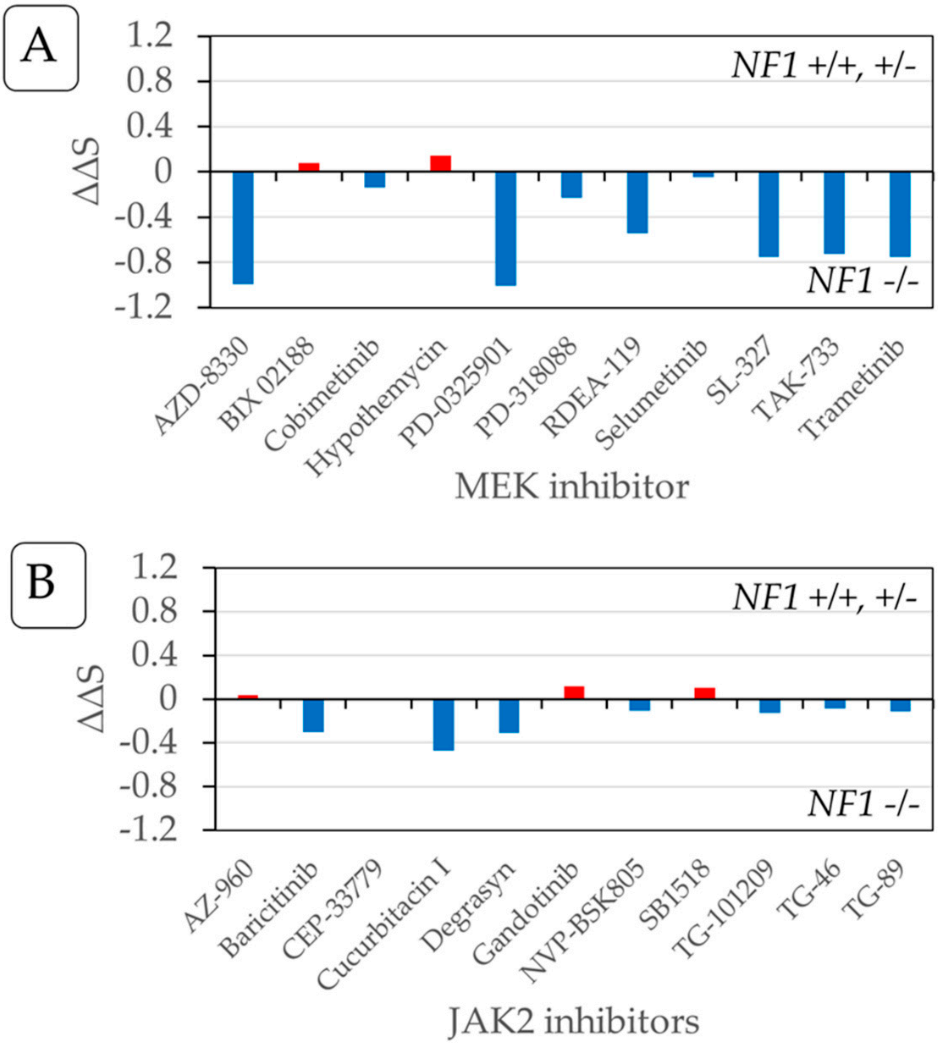

Supplement Figure S1. *ΔΔS* outcomes from a series of compounds (**A**) targeting MEK, expected to be sensitive, and (**B**) targeting JAK2, expected not to be sensitive. Both (**A**) and (**B**) use a group of control (*NF1*+/+, +/-) cell lines (ipNF02.3 2λ, ipNF02.8, and ipnNF95.11C) and were compared against plexiform neurofibroma (*NF1*-/-) cell lines (ipNF05.5-MX, ipNF06.2A, ipNF95.11b C/T, and ipNF95.6). HFF (human foreskin fibroblast) was used as the common reference cell line (**A**) and (**B**). Due to missing values primarily in HFF of the original data set not all compounds in each inhibitor set could be presented. Notice a pronounced lower magnitude of effect across available JAK2 inhibitors, relative to MEK inhibitors.

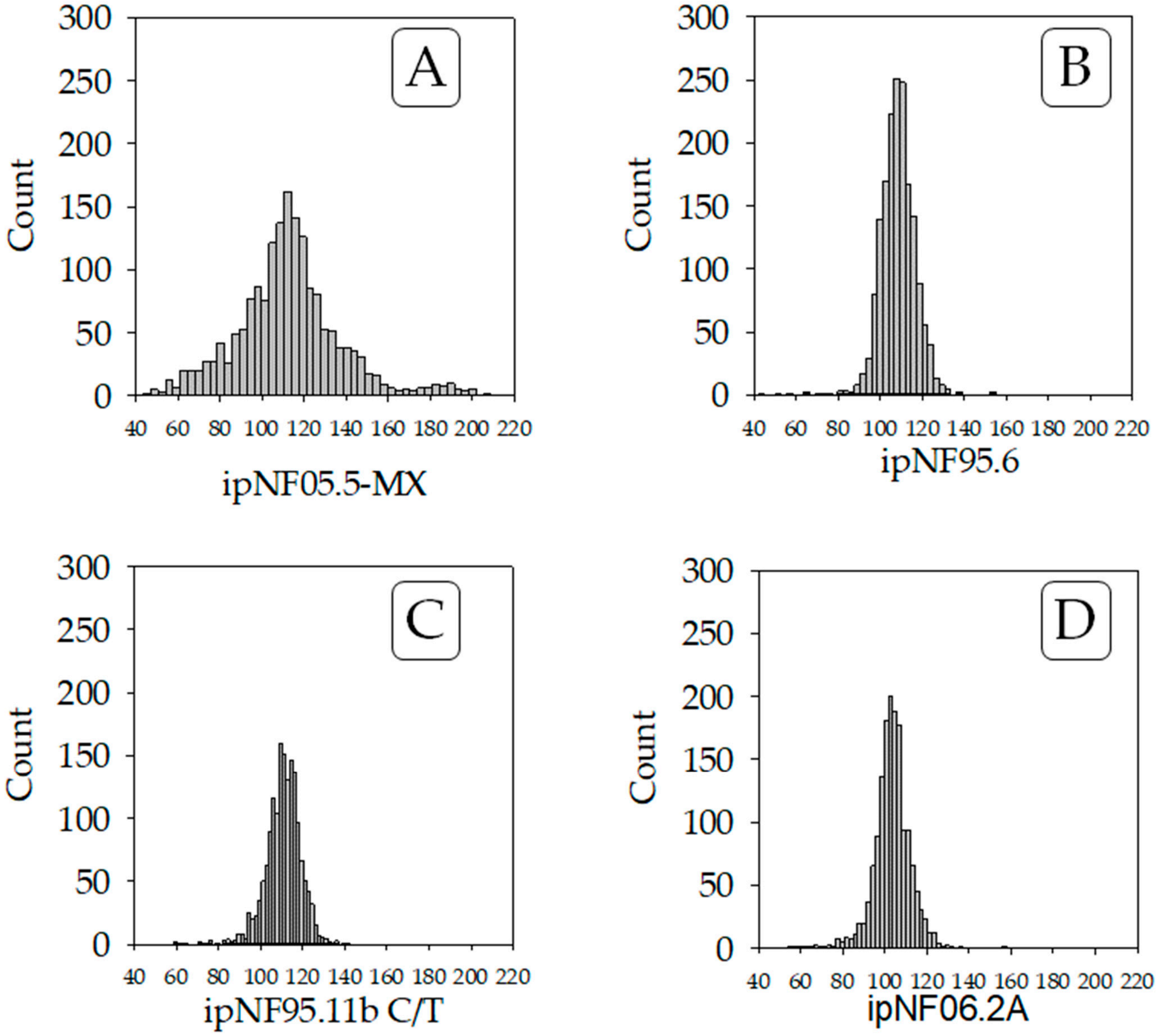

Supplement Figure S2. Histogram of maximum (upper) asymptotes for all tested compounds by target plexiform neurofibroma cell line. The *y*-axis describes the number (count) of compounds in each bin while the *x*-axis describes the response as % of the DMSO control taken for the asymptote from the concentration response curve. All the histograms **(A-D)** demonstrated a bell shaped curve with broadly similar centers (103-, 109-, 111-, and 112 % of the DMSO control value for ipNF06.2A, ipNF95.11b C/T, ipNF95.6, and ipNF05.5-MX, respectively). For each histogram the *x*-axis range was set to 40-220 % of the DMSO control with individual responses allocated to 50 bins. Note the general similarity of the centers and overall histogram shape, although ipNF05.5=MX has a more extensive spread of the histogram. The ipNF05.5-MX is a mixed clone cell line and that may also contribute to its variability.

Drug Response Evaluation and Assessment (DREA) Web Tool

An exploratory Drug Response Evaluation and Assessment (DREA) Web Tool was developed in the Python programming language for our methods. This tool can be accessed at https://nf.mocomakers.com and it includes data visualizations, our derived fields, as well as additional filters and cell lines for expanded investigation potential. For more tips on using this tool, please see: https://github.com/MoCoMakers/nf_streamlit/wiki.
